# Potential of mesenchymal stem cells as topical immunomodulatory cell therapies for ocular surface inflammatory disorders

**DOI:** 10.1002/sctm.20-0118

**Published:** 2020-09-08

**Authors:** Lydia J. Beeken, Darren S.J. Ting, Laura E. Sidney

**Affiliations:** ^1^ Academic Ophthalmology, Division of Clinical Neurosciences University of Nottingham, Queens Medical Centre Campus Nottingham UK

**Keywords:** cell therapy, cornea, inflammation mediators, mesenchymal stem cells, regenerative medicine

## Abstract

Ocular surface inflammatory disorders (OSIDs) are a group of highly prevalent, heterogeneous diseases that display a variety of aetiologies and symptoms and are risk factors for serious complications, including ocular and cornea impairment. Corneal inflammation is a common factor of all OSIDs, regardless of their cause or symptoms. Current medications include over‐the‐counter lubricating eye drops, corticosteroids, and ciclosporin, which either do not treat the corneal inflammation or have been associated with multiple side effects leading to alternative treatments being sought. Regenerative medicine cell therapies, particularly mesenchymal stem cells (MSCs), have shown great promise for immunosuppression and disease amelioration across multiple tissues, including the cornea. However, for successful development and clinical translation of MSC therapy for OSIDs, significant problems must be addressed. This review aims to highlight considerations, including whether the source of MSC isolation impacts the efficacy and safety of the therapy, in addition to assessing the feasibility of MSC topical application to the cornea and ocular surface through analysis of potential scaffolds and cell carriers for application to the eye. The literature contains limited data assessing MSCs incorporated into scaffolds for corneal administration, thus here we highlight the necessity of further investigations to truly exploit the potential of an MSC‐based cell therapy for the treatment of OSIDs.

## Graphical abstract

Cell therapies are a potential candidate for the topical treatment of ocular surface inflammatory disorders (OSIDs). This review highlights important factors for translational consideration, including optimal cell type, the source of the cell and the application to the ocular surface. Ultimately, through combining potent immunomodulatory cells with functional scaffolds, an efficacious, topical cell therapy for OSIDs could be engineered.

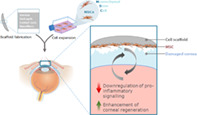

**Significance statement**

This is the first review focusing on the potential of engineering mesenchymal stem cell (MSC) therapies that can be applied topically to the ocular surface, in order to treat inflammatory disorders that cannot be managed through steroids or other means. This study aims to highlight different considerations, including whether the source of MSC isolation may impact the efficacy and safety of the therapy, in addition to assessing the feasibility of topical stem cell application to the ocular surface through analysis of potential scaffolds.

## 1 INTRODUCTION

**FIGURE 1:**
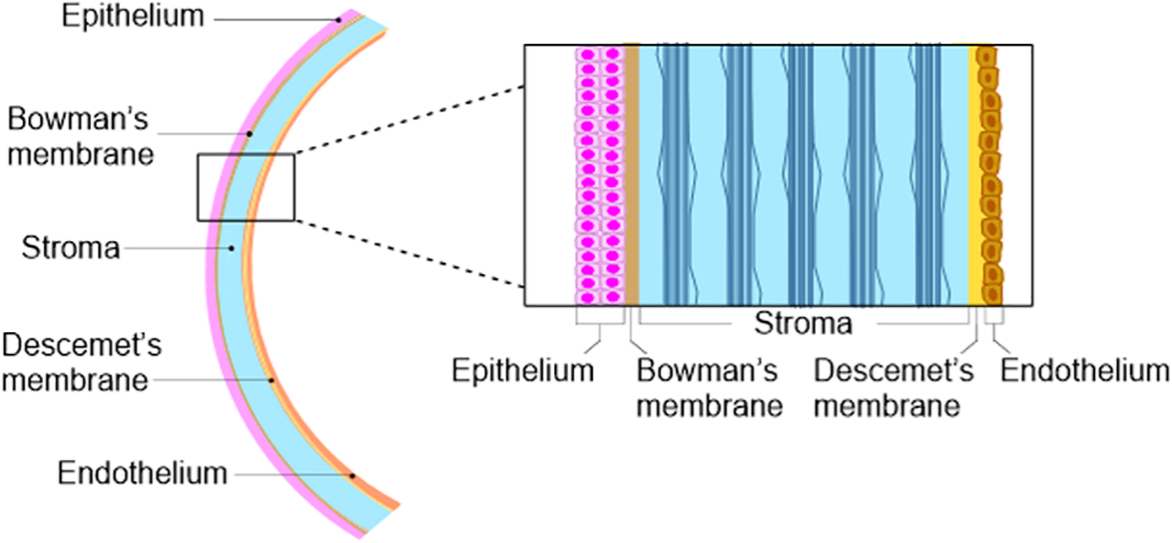
The structure of the cornea. Working from the ocular surface anterior to posterior, the cornea is made up of an epithelium; Bowman's membrane; stroma; Descemet's membrane, and endothelium

The cornea is a highly organized, transparent tissue at the ocular surface. It is comprised of three main cellular layers: the epithelium, the stroma containing the keratocytes, and the endothelium, separated by the Bowman's membrane and Descemet's membrane, respectively^1^ (Figure 1). Coating the outer mucosal surface of the cornea is the tear film, a thin, liquid layer,^2^ mainly constituted of mucin and lipid. As the cornea is avascular, the tear film plays a vital role in the supplementation of nutrients and oxygen, as well as the expulsion of waste such as epithelial debris, foreign bodies, and toxins. Interactions between the ocular surface and the tear film allow for a smooth optical surface, correct functioning of limbal epithelial cells and protection from mechanical and microbial insults.^3^ Additionally, healthy corneal tissue is maintained through tight immunoregulatory mechanisms at the ocular surface, modulated by both the innate and adaptive immune systems.

Ocular surface inflammatory disorders (OSIDs) occur when the tightly regulated homeostasis at the ocular surface is disturbed, and encompass a range of heterogeneous diseases with a variety of aetiologies and symptoms, where inflammation plays a critical role in pathogenesis.^4^ Dry eye disease (DED), meibomian gland dysfunction (MGD), allergic eye diseases, cicatricial conjunctivitis, chemical eye burn, trauma, iatrogenic insult following corneal and/or refractive surgery, and contact lens‐related complications are the common examples of OSIDs that are frequently encountered and managed in clinical practice.

OSIDs are highly prevalent in the general population. For example the global prevalence of DED has been estimated at around 5% to 50% depending on the diagnostic criteria and study population.^5^ MGD, a major contributor to evaporative DED, has been shown to cause a myriad of negative impacts on the ocular surface including heightened inflammation, oxidative stress, tear hyperosmolarity, and increased corneal epitheliopathy.^6^ These diseases often serve as an important risk factor for major ocular surface complications including infectious keratitis, corneal vascularization, opacity, visual impairment, corneal melt, and perforation.^7^, ^8^, ^9^ In addition, OSIDs are regularly associated with pain and irritation, causing a considerable reduction in the patient's quality of life, activities in daily living, and work productivity.^10^ Irrespective of their source, insult to the cornea ultimately results in a vicious cycle, where chronic irritation activates an immune response, augmenting the irritation.^4^

Currently, treatments include over‐the‐counter lubricating eye‐drops to alleviate disease symptoms, and corticosteroids to ameliorate the inflammation. However, these treatments require long‐term topical application, multiple times a day (every hour), placing high demand on patient compliance and interfering with their day‐to‐day life. Furthermore, corticosteroids have been linked to severe adverse effects including increased risk of infectious keratitis, inhibition of corneal wound healing, raised intraocular pressure, and cataracts.^11^, ^12^ Ciclosporin serves as a valuable steroid‐sparing immunomodulatory agent for managing a range of OSIDs, although side effects are common.^13^ Lifitegrast, a recent FDA approved drug, represents another useful topical anti‐inflammatory treatment for DED. However, both ciclosporin and lifitegrast are associated with a high rate, up to 70%, of side effects, including burning sensation, itching, and blurred vision, among others.^14^

Because of the abundance of therapeutic factors possessed by human stem cells, regenerative medicine may hold the key to developing a superior treatment to alleviate OSIDs. This review outlines the process required for the application of stem cell therapy for OSIDs, through assessing optimum cell type and delivery method to the ocular surface. Here, we focus on the use of mesenchymal stem cells (MSCs) due to their well‐accepted immunomodulatory properties and suggest that applying the cells topically, via a removable substrate or scaffold, may offer the most convenient and efficacious therapy.

## 2 POTENTIAL SOURCES OF STEM CELLS FOR IMMUNOMODULATION OF THE INJURED OCULAR SURFACE

Inflammation is recognized as a significant feature in the etiopathophysiology of OSIDs, therefore stem cells with efficacious anti‐inflammatory properties would be optimal for successful treatment. Limbal epithelial stem cell transplantation (LSCT) and cultivated corneal epithelial (CCE) sheets have shown promising therapeutic results for restoring a normal corneal epithelial phenotype in patients with severe chemical injury and dry eye.^15^, ^16^ However, the primary utilization of LSCT and CCE is to generate an entire new epithelial layer in situ or in vitro, respectively, rather than for their immunosuppressive capacity, used predominantly in cases where injury has resulted in a limbal epithelial stem cell deficiency (LSCD). Their incapacity to suppress inflammation is supported by data demonstrating contraindications of LSCT in the presence of active inflammation in bilateral diseases, including Stevens‐Johnson syndrome, ocular cicatricial pemphigoid, and graft vs host disease (GVHD). In fact, the failure of LSCT is often accredited to sites of active inflammation creating a toxic microenvironment at the ocular surface.^17^ Although these techniques have proven, in some cases, successful to treat injuries such as chemical burn, which are associated with high levels of inflammation, it is likely that some of the immunosuppression was governed and achieved by the immune‐modulating, amniotic membrane (AM) scaffold the cells were applied with.^15^, ^16^ As the pros and cons of LSCT have been covered in previous reviews,^17^, ^18^ we wish in this review to highlight alternative sources of stem cells that could be considered for novel regenerative medicine therapies.

Differentiating induced pluripotent stem cells (iPSCs) into immune‐mediating cells, such as regulatory T cells,^19^ holds the potential to improve the inflammatory symptoms of OSIDs. However, this therapeutic strategy is limited by the high tumorigenic potential, cost, and regulation associated with the generation and application of iPSCs.^20^

MSCs are best known in regenerative medicine for their ability to modulate both the innate and adaptive immune systems,^21^ suggesting a potential use for the treatment of inflammation in OSIDs. Their capacity to reduce inflammation has been assessed in vitro and in vivo on multiple tissues, including the kidney, heart, cartilage, liver, brain, skin, and cornea,^22^ with preclinical success demonstrated by their current use in clinical trials.^23^ MSCs encompass a group of fibroblast‐like, multipotent progenitor stromal cells, defined initially by their capacity to differentiate into osteoblasts, adipocytes, and chondrocytes,^24^ however MSCs are now utilized primarily for the ability to elicit a therapeutic response through communication with target tissue cells.

## 3 DIRECT COMMUNICATION OF MSCs AND TARGET CELLS

Limited evidence has demonstrated that MSCs can interact with the target tissue directly via cell‐cell contacts such as gap junctions and tunneling nanotubes.^25^ This has been demonstrated in cardiac tissue, where the respiratory chain in myocytes was salvaged through mitochondrial transfer. Although not investigated in the literature, hypothetically this mechanism could restore cells at the ocular surface and is therefore an area with potential for future exploration.

## 4 PARACRINE SIGNALLING OF MSCs AND POTENTIAL EFFECT ON CORNEAL IMMUNOMODULATION

The main interest surrounding MSCs has shifted to their paracrine function, as a positive therapeutic response can be achieved irrespective of whether the cells reach the target organ.^26^ There is an abundance of data demonstrating MSC secretion of anti‐inflammatory factors, cell‐mobilization factors, and growth factors in response to inflammatory mediators.^27^

**FIGURE 2:**
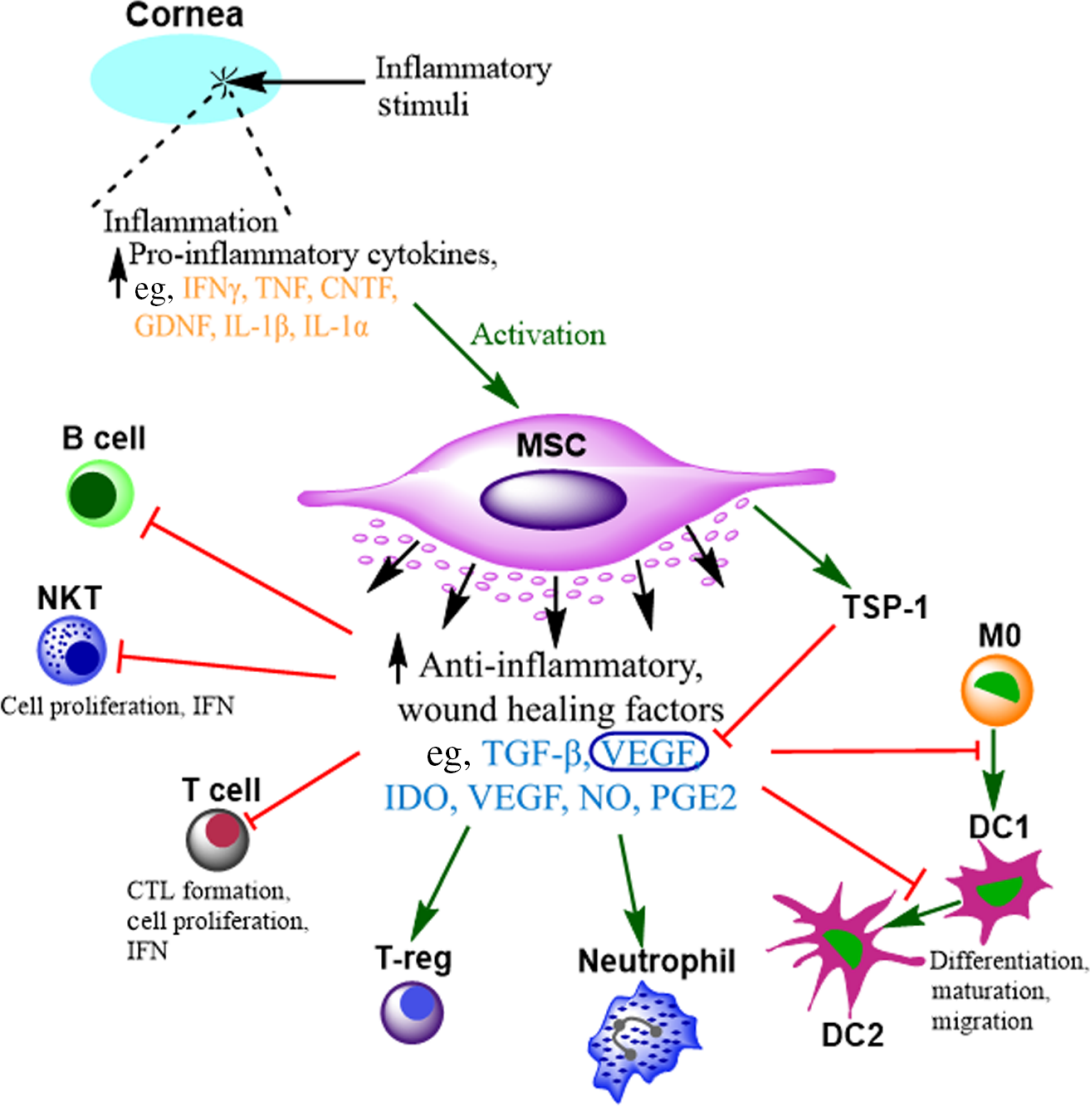
Immunomodulation by mesenchymal stem cells (MSCs). Inflammatory stimuli at the ocular surface results in an increase in pro‐inflammatory factors, for example interferon‐γ (IFN‐γ), tumor necrosis factor (TNF), ciliary neurotrophic factor (CNTF), glial cell‐line derived neurotrophic factor (GDNF), and interleukins (IL) 1β and 1α. These factors can activate and stimulate any applied MSCs to secrete immunomodulatory factors including transforming growth factor β (TGF‐β), IL‐10, indoleamine 2,3‐dioxygenase (IDO), nitric oxide (NO), prostaglandin E2 (PGE2), and vascular endothelial growth factor (VEGF). This can result in the inhibition (red line) of proliferation and function of T and B lymphocytes, natural killer T cells (NKTs), and dendritic cells (DCs), however, can preserve neutrophil viability through apoptosis inhibition. MSCs also stimulate (green arrow) the upregulation of thrombospondin‐1 (TSP‐1) in the cornea, which inhibits VEGF and prevents angiogenesis

Stimulation of MSCs with interferon‐y (IFN‐y) has been studied abundantly in the literature, demonstrating activation of the IFN‐γ‐Janus kinase (JAK)‐signal transducer, and activator of transcription (STAT) 1 pathway^28^ leading to the secretion of indoleamine 2,3‐dioxygenase (IDO), a tryptophan catabolizing enzyme commonly directly correlated with the immunomodulatory potency of MSCs.^29^ MSC activation has also been investigated with pro‐inflammatory cytokines tumor necrosis factor‐α (TNF‐α) and interleukins (IL)‐1α/‐1β, leading to upregulation of transcription factors including NFkB, and the secretion of several factors including transforming growth factor‐β, ciliary neurotrophic factor, glial cell line‐derived neurotrophic factor, interleukins‐1β, ‐6, ‐8, and ‐10, nitric oxide (NO), hepatocyte growth factor (HGF), and vascular endothelial growth factor (VEGF) (Figure 2).^30^

Using paracrine signaling, MSCs can ultimately suppress the activation and function of various cells within the adaptive and innate immune systems, including T and B lymphocytes, macrophages, natural killer cells, neutrophils, and dendritic cells. Multiple corneal and ocular surface studies have demonstrated the reduction of inflammatory factors following MSC administration in vitro and in vivo,^31^, ^32^ in addition to their capacity to inhibit allergy driven disease, such as allergic conjunctivitis, through COX‐2‐dependent anti‐allergic mechanisms.^33^

An initial consideration regarding the use of MSCs for OSIDs is the relationship between secreted growth factors and angiogenesis. In ischemic cardiac tissue, MSCs promote neovascularization through the upregulation of VEGF.^34^ Ocular angiogenesis is a lead factor of blinding eye diseases including retinal disease, such as age‐related macular disorder (AMD), stimulated by an increase in, VEGF.^35^ Conversely, MSCs have shown the opposite effect on neovascularization when applied to corneal injury induced by chemical burn. One study demonstrated downregulation of VEGF and significant reduction of neovascularization in the MSC‐treated cornea.^36^ This could be attributed to MSC induced upregulation of thrombospondin‐1, a VEGF inhibitor^37^ and signifies the importance of the microenvironment on MSC behavior.

HGF has also been implicated as a fundamental factor in immunomodulation, secreted by MSCs stimulated with IL‐1ß.^38^ HGF alone is powerful enough to suppress antigen presenting cell activation and to limit the generation of Th1 cells in the lymphoid tissue. Topical HGF application significantly reduced the rejection of corneal grafts in a murine model of GVHD, through suppression of immune cell infiltration, and has the potential to maintain and restore corneal transparency through the inhibition of α‐SMA and its inducer TGF‐ß.^31^, ^39^

Other key anti‐angiogenic molecules secreted by MSCs include TNF‐α stimulated gene/protein (TSG‐6), demonstrated as vital in the inhibition of neovascularization, and suggested to function through the inhibition macrophage infiltration and the induction of apoptosis of vascular endothelial cells.^40^ As well as macrophages, TSG‐6 has been demonstrated to suppress activation and infiltration of neutrophils following chemical and mechanical corneal injuries,^41^ making it a potent modulator of both angiogenesis and inflammation.

An alternative method to exploit this paracrine signaling mechanism of MSC to treat OSIDs would be through harvesting extracellular vesicles from the MSC for therapeutic application.^42^ The potent therapeutic factors of MSCs packaged in small vesicles could help to overcome the safety and regulatory hurdles of cell application and have shown potential in corneal wound healing and immunomodulation in vivo.^43^

Fully elucidating the pathways and interactions of different MSCs and the corneal microenvironment will help to increase the safety profile and therapeutic value of these cells for both tissue regeneration and inflammation suppression, highlighting the necessity to explore different MSC sources.

## 5 MSC SOURCE

It is of utmost importance to consider MSC source, both tissue and donor (autologous or allogeneic). Although MSCs have previously been claimed as immune‐privileged because of their lack of expression of Major Histocompatibility Class (MHC) II proteins and co‐stimulatory molecules B7 and CD40 ligand,^44^ immune rejection of MSCs derived from allogeneic sources has proven a major therapeutic challenge for application to a wide variety of conditions.^45^ Similarly, the ocular microenvironment has been claimed to be immune‐privileged, with original accounts demonstrating placement of a foreign antigen in the eye did not elicit an immune response.^46^ Although GVHD is a contraindication of an ocular allogeneic stem cell transplant in approximately 40% to 60% of patients,^47^ the immunomodulatory properties of MSCs may give them additional protection, even if from an allogeneic source, with reports of multiple clinical trials using MSCs to both prevent and treat GVHD.^48^ Although allogeneic cell therapy is beneficial for the manufacturing of the therapy, potential adverse effects of foreign cells are vital to consider.

MSCs can be isolated from most tissues in the body and cultured in vitro, however they do not all possess the same properties. For example, literature demonstrating MSC secretion of the anti‐inflammatory cytokine, IL‐10, is highly contradictory, and could be because of the source of the cells.^49^ For successful translation to clinic, it is important that multiple sources of MSCs are explored, to develop the most efficacious and cost‐effective treatment for OSIDs.

### 5.1 Bone marrow‐derived MSCs (BM‐MSCs)

Bone marrow is the most investigated source of MSCs in OSID cell therapy research. BM‐MSCs have demonstrated efficacy for immunoregulation and disease amelioration in multiple in vivo OSID models with different administration routes. These include animal models of chemical burns^36^, ^50^ and inflammation‐induced dry eye.^51^ However, a major limitation includes the invasive and painful procedure to isolate the bone marrow, where only 0.001% to 0.01% of the cells will constitute MSCs.

### 5.2 Adipose‐derived MSCs (AD‐MSCs)

AD‐MSCs have similar levels of surface antigen expression, differentiation ability, and immunosuppressive activity as BM‐MSCs,^52^ and can be isolated in abundance because of plentiful, accessible sources, which can generate a higher yield of 100 to 1000 cells per gram of adipose tissue. However, data demonstrating their efficacy for corneal regeneration are scarce and conflicting. Fuentes‐Julián et al^53^ found that application of AD‐MSCs to a rabbit model of corneal allograft rejection increased inflammation levels. In contrast, AD‐MSCs have shown efficacious effects on numerous other organs including the liver and brain,^54^, ^55^ achieved through suppression of the immune response. A recent study which compared them directly to BM‐MSCs found a reduced capacity for corneal wound healing in vitro.^56^ Further research is required to determine whether AD‐MSCs have translational properties across tissues, or to understand their differential behavior in the corneal allograft rejection model. Additionally, major safety concerns regarding the use of AD‐MSCs were recently uncovered following intravitreal injection of autologous AD‐MSCs in a clinical trial for non‐vascular AMD. Although a retinal disorder, it is important to note the trial induced vision loss because of retinal detachment and increased intraocular pressure following MSC administration.^57^

### 5.3 Corneal‐derived MSCs (C‐MSCs)

Each MSC niche is different, leading to a risk of cells exhibiting unexpected behavior when transplanted into a separate tissue. Therefore, there may be therapeutic benefits to transplanting MSCs already accustomed to the corneal microenvironment, back onto the ocular surface. It has been demonstrated that when isolated and expanded in vitro, keratocytes from the corneal limbal stroma assume an MSC phenotype.^58^, ^59^, ^60^ Furthermore, these C‐MSCs show anti‐inflammatory potential when cocultured with injured corneal epithelial cells,^32^ can reduce corneal scarring after wounding,^61^ and express specific markers of the cornea when other MSC types do not.^62^, ^63^ C‐MSC secreted exosomes have also demonstrated the capacity to accelerate corneal epithelial wound healing.^43^

### 5.4 MSCs from other sources

Dental pulp (DP) and umbilical cord blood (UCB) are alternative sources of MSCs. DP‐MSCs display similar marker characteristics and differentiation potential to the aforementioned MSCs, in comparison to UCB‐MSCs which show higher levels of proliferation, more potent levels of immunomodulation, and lower levels of senescence.^52^ Although limited research applies these cells to the cornea, an ex vivo study has demonstrated the capacity of DP‐MSCs to enhance repair and regeneration of human corneal epithelium, immature DP‐MSCs have shown efficacy in vivo for LSCD, causing decreased corneal opacity and neovascularization,^64^ in addition to both directly and indirectly inducing corneal epithelial wound healing in vitro,^65^, ^66^ highlighting their potential as a therapeutic agent.

## 6 EFFECT OF CULTURE, PASSAGE, AND PRIMING OF MSCs

The effect of culture and passage must be balanced when considering MSCs as a therapeutic agent. Optimally, the maintenance of MSC phenotype and behavior is vital, however the ability to culture cells to high passage numbers allows greater opportunity for allogeneic scale‐up. in vitro passage investigations have shown that ageing MSCs are subject to morphological changes and reduced immunomodulatory capacity with a significant reduction in release of trophic factors such as VEGF,^67^, ^68^ lead to the use of innovative culture techniques such as the Quantum hollow fiber bioreactor, to culture greater number of cells without adverse changes.^69^ Optimization of culture medium should also be performed as different media have been shown to affect the phenotype of initially identical cell populations.^70^

Priming, or “licensing” of the cells, with in vitro application of cytokines such as IFN‐y has been shown to improve immunosuppressive capacity and pharmaceutical utility.^71^ Although the mechanisms are not fully elucidated, suggested explanations include the upregulation of IDO, the clustering of MHC and co‐inhibitory molecules, and epigenetic changes.^72^, ^73^ Additionally, priming the cells through hypoxia treatment and activation of the MSC nucleotide binding domain, as well as techniques including gene modification existing to improve therapeutic potential.^74^

## 7 APPLICATION OF MSCs TO THE OCULAR SURFACE: TOPICAL VS ALTERNATIVE METHODS

A comparison of studies demonstrating MSC efficacy in various ocular surface disease models, using different delivery mechanisms can be found in Table 1. In contrast to developing stem cell therapies for internal organs, the location of the ocular surface makes it an ideal candidate for the noninvasive topical application of stem cells. The advantages of topical application of MSCs, in a similar manner to that discussed for skin healing,^89^, ^90^ include: the ability to deliver a concentrated population of cells to a small area, without relying on cell homing mechanisms; the immediate delivery of paracrine signaling molecules to the target area, allowing for more rapid healing; the potential ability to remove the cells after healing if adhered to the delivery vehicle, potentially avoiding allogeneic rejection; and the less invasive nature of the treatment, delivered within a clinic setting rather than surgically.

**TABLE 1:** Comparison of studies demonstrating mesenchymal stem cell (MSC) efficacy in various ocular surface disease models, using different delivery mechanisms

MSC source	Delivery mechanism	Procedure	Cell passage	Animal model	Study length	Key findings	Ref.
Human AD‐MSCs	Topical	2.5E+05 cells seeded on amniotic membrane; eyelids sutured	3/4	Rabbit partial and total LSCD	11 weeks	MSCs migrated to inflamed tissues, reduced inflammation, inhibited neovascularization, and corneal opacification, and expressed CK3 in the corneal epithelium, demonstrating partial restoration of epithelial phenotypes	^75^
Human AD‐MSCs	Topical	1.25E+05/mm^2^ cells seeded on a scleral contact lens	3	Rabbit severe acute alkaline burn	4 weeks	MSCs prevented corneal melting and symblepharon, reduced the inflammatory and fibroblastic response, and significantly reduced epithelial defects	^76^
Human BM‐MSCs	Topical	1E+05 cells/cm^2^ seeded on amniotic membrane and cultured to 90% confluence. Eyelids sutured for 10 day	1	Rat corneal chemical burn	4 weeks	MSCs enhanced repairmen of injured ocular surface and epithelial integrity. They inhibited inflammation and inflammation‐induced neovascularization, decreasing levels of IL‐2 and CD45, therefore improving transparency	^77^
Mouse BM‐MSCs	Topical	4E+04 cells on polyamide 6/12 nanofiber scaffold. Eyelids sutured closed. Also seeded with LESCs	Unknown	Mouse corneal mechanical injury	2 weeks	MSCs significantly reduced immune response, through suppression of IFN‐y, iNOS, and IL‐2 gene expression in local corneal cells	^78^
Rabbit BM‐MSCs	Topical	Unknown cell dose seeded in a fibrin gel, sutured to corneal surface. Eyelids sutured for 7 days	Unknown	Rabbit corneal alkali burn	4 weeks	MSCs did not improve corneal epithelium integrity, neovascularization, or corneal opacity. They expressed CK3, demonstrating differentiation into corneal‐like cells	^79^
Rabbit BM‐MSCs and AD‐MSCs	Topical	3E+05 cells seeded on PLA nanofiber scaffold, sutured to the conjunctiva. Eyelids closed	3	Rabbit corneal alkali burn	15 days	MSCs caused suppression of MMP9 and iNOS, reduced levels of αSMA, TGF‐B, and VEGF, leading to reduced corneal opacification, neovascularization, and corneal thickness	^50^
Rat MSC line. Source unknown	Topical	2E+06 cells in media applied for 2 hours a day for three consecutive days. Eyelids sutured	Unknown	Rat corneal chemical burn	3 weeks	MSC anti‐inflammatory potency through IL‐6 suppressing maturation of DCs, and anti‐angiogenic through upregulation of TSP‐1	^37^
Human and mouse C‐MSCs	Topical	5E+03 cells in a fibrinogen (Ethicon) gel, eyelids sutured	3	Mouse pathologic corneal vascularization	3 days	Inhibition of corneal neovascularization, likely through secreted sFLT‐1 and PEDF	^80^
Human immature DPSCs	Topical	Cell sheet held in place with sutured amniotic membrane	6 to 7	Rabbit mild and severe chemical induced LSCD	3 months	MSCs lead to reconstruction of the corneal epithelia in the mild model, but not severe. Cells in both models adopted an epithelial‐like phenotype	^64^
Rabbit limbal‐MSC	Topical	0.5E+05 cells/cm^2^ on human AM. Also seeded with LESCs. AM sutured into place	3/4	Rabbit epithelial debridement and limbal keratectomy	12 weeks	MSCs promoted epithelialization, however neovascularization was seen when L‐MSC were applied without LESC. Cell did not migrate into the healing epithelium	^81^
Human BM‐MSCs	Intravenous injection	1E+06 cells in balanced salt solution	2	Mouse suture induced corneal neovascularization	1 week	MSCs reduced neovascularization, through a TSG‐6 dependent mechanism. They reduced inflammation through reduction of IL‐1B, IL‐6, and TNF‐a and suppression of infiltrating immune cells	^40^
Mouse BM‐MSCs	Intravenous injection	5E+05 cells in saline. Eyelids sutured closed for 3 days	2	Mouse corneal transplant model	2 months	MSCs inhibited corneal leukocyte infiltration, maturation of APCs and generation of Th1 cells to promote graft survival. Data show HGF as key paracrine factor	^38^
Mouse BM‐MSCs	Intravenous injection	5E+05 cells in saline	2	Mouse corneal mechanical injury	3 days	MSCs lead to increased levels of HGF at the ocular surface, which helped to restore corneal transparency and suppress TGF‐B‐induced α‐SMA expression	^39^
Mouse BM‐MSCs	Intravenous injection	1E+06 cells	5 to 6	Mouse corneal transplant model	2 weeks	MSCs homed directly to the inflamed ocular surface, inhibited APC maturation, suppress allosensitization, and promote allograft survival	^31^
Rat BM‐MSCs	Intravenous injection	1E+06 cells in PBS	2	Rat high‐risk corneal transplant model	37 days	MSCs increased rejection‐free survival, reducing inflammation through increasing regulatory T cells and release of immunomodulatory mediators including PGE2	^82^
Rabbit AD‐MSCs	Intravenous injection	2E+06 cells in HBSS. 4 injections; D‐7, D0, D3, and D14‐15	3 to 4	Rabbit high‐risk corneal allograft rejection model	19 days	MSCs did not home to cornea or engraft. MSCs increased edema and neovascularization and had no effect on infiltration of immune cells	^53^
Human BM‐MSCs	Subconjunctival injection	2E+05 cells in PBS	3	Mouse GVHD	18 days	MSCs did not engraft but prevented T lymphocyte infiltration and reduced inflammatory gene markers *TNF*, *PAX6*, and *Sprr1b* and reduced keratinization of the cornea	^83^
Mouse BM‐MSCs	Subconjunctival injection	5E+04 cells in PBS	3 to 5	Diabetic mouse model of corneal epithelial injury	3 days	Homing of MSCs to wound edge of cornea, with TSG‐6 secretion responsible for enhanced wound healing, increased epithelial stem cell proliferation, and reduction of inflammatory infiltrates and inflammatory markers; MPO, TNF‐α, and IL‐1β	^84^
Rat BM‐MSCs	Subconjunctival injection	2E+06 cells in PBS. Used polysaccharide hydrogel as bandage	3	Rat corneal alkali burn	4 weeks	MSCs promoted epithelial recovery, corneal clarity, reduced neovascularization, and reduced MIP‐1a and MCP‐1. All results were enhanced with hydrogel	^85^
Human and mouse BM‐MSCs	Periorbital injection	1E+03 or 1E+05 cells in balanced salt solution	2	Mouse inflammation‐induced dry eye	1 week	MSCs did not engraft, but increased tear production, reduced CD4+ IFN‐γ secreting cell infiltration and restored goblet cells in the conjunctiva	^51^
Human UC‐MSCs	Multiple	2E+06 cells in PBS	5	Rabbit corneal alkali burn	4 weeks	MSCs lead to reduced neovascularization, corneal opacification, and VEGF and α‐SMA in the cornea. They also resulted in increased re‐epithelialization and proliferation of keratocytes	^86^
Mouse BM‐MSCs	Multiple	5E+05 cells in PBS	3	Mouse corneal mechanical injury	4 days	MSCs administered through intravenous and subconjunctival injection significantly reduced inflammation, corneal opacity, fibrosis, and restored epithelial integrity and tissue architecture. No significant difference observed for topical and intraperitoneal administration	^87^
Human UC‐MSC	Multiple	2E+04 cells in alpha‐MEM (intrastromal injection) 2E+04 cells in a fibrin gel carrier	Unknown (after P4)	Mouse keratectomy wound	2 weeks	MSCs increased corneal transparency and increase collagen fiber organization	^88^

Topical delivery of MSCs has potential of enhanced therapeutic capacity, supported by in vitro studies showing increased suppression of T‐lymphocytes and corneal wound healing with direct MSC contact, compared to MSC paracrine factors alone in culture medium.^91^, ^92^ When applied systemically, MSCs often become entrapped in the pulmonary circulation, and although still generate ameliorating effects on distant organs through paracrine signalling,^26^ may be more efficacious at the site of healing.

For the eye, subconjunctival injection has demonstrated success at ameliorating disease in multiple ocular surface disorder models, including GVHD^83^ and in corneal injury,^87^ where subconjunctival injection was deemed more effective than systemic and topical application. However, it is important to note that in this study, the cells applied topically were not incorporated into a scaffold to hold them in place and would likely have been expelled through lachrymation and blinking. Consequently, for topical application of MSCs at the site of injury to be efficacious, a cell carrying scaffold is required to ensure persistence of cells placed directly into the toxic microenvironment of an injured ocular surface.

Although, potentially overlooked, the choice of delivery substrate/scaffold may have a significant impact on the eventual therapy, with evidence demonstrating a fivefold increase of factors such as HGF and ICAM‐1 when MSCs were cultured on 3D fiber matrices compared to 2D culture dishes, promoting faster epithelialization and reduced scarring.^93^

### 7.1 Potential substrates and scaffolds for topical application of MSCs to the ocular surface

AM is often the substrate of choice for any delivery of cells to the ocular surface, due to its long history of use within the field. AM is the translucent, inner fetal layer, lining the amniotic cavity with demonstrated low immunogenic, anti‐scarring, and anti‐inflammatory properties.^94^ For example, AM alone has the potential to induce rapid apoptosis in adhered, inflammatory cells, including T‐lymphocytes and macrophages in corneas of herpetic stromal keratitis mouse models.^95^ AM can be optimally preserved through freezing or drying to maintain the structural and biochemical properties,^96^ before cells can be seeded and the structure glued or sutured into place.^97^ Alternative, sutureless methods have been investigated, such as application via ProKera,^98^ or application using bandage contact lenses (CLs).^99^ AM in combination with MSCs has been shown to provide a beneficial, additive effect, demonstrated in a chemical burn rat model where injury was significantly improved.^77^ However, inter and intra donor variation and risk of disease transmission represent a lack of standardization.

Alternative to AM, the use of both natural and synthetic hydrogels may offer more consistency, easier manufacturing, and potentially simpler application, as they can be manufactured as soft CLs. Hydrogels are 3D, polymer networks, with elastic properties and open systems for substance exchange.^100^ Most research investigating stem cell‐hydrogel applications are designed with the primary intention to bioengineer an entire new epithelial layer for transplantation to treat LSCD. However, Gu et al^79^ incorporated MSCs with a fibrin hydrogel for ocular surface transplantation and demonstrated improvement of corneal injury. It is likely the therapeutic effect seen in this study was a result of MSC immunomodulation, supported by data demonstrating that MSCs have the capacity to secrete paracrine signals when incorporated into a hydrogel.^101^ Ke et al also found that combination of a topical polysaccharide hydrogel and subconjunctival injection of BM‐MSCs performed additively to enhance corneal epithelial cell recovery and corneal clarity in a rat model of alkali burn,^85^ reinforcing the idea that the choice of substrate if as important as the stem cell.

Synthetic hydrogel bandage CLs are currently used to protect the corneal surface in combination with the delivery of pharmacological or biological therapeutics.^102^ Most are composed of siloxane hydrogel,^103^ and hold desirable qualities, while the absence of protein reduces the risk of allogeneic rejection or disease transmission,^104^ and their shape allows self‐maintenance on the cornea. To avoid the undesirable effects of corneal epithelial cell attachment and protein fouling when placed on the ocular surface, CL materials rarely contain cell adhesion motifs, and consequently must be functionalized to behave as a cell delivery device; these can be provided by integrin binding sites from serum, 3T3 feeder layers, and surface plasma polymerization with acid groups.^105^, ^106^, ^107^

Three‐dimensional scaffolds produced via electrospinning have a large surface area, with the nanofibers arranged to imitate extracellular matrix proteins. MSCs have been demonstrated to attach and proliferate effectively on these scaffolds, and when applied to the cornea aid healing and regeneration.^78^, ^108^ Further modification to allow for the possibility of cell detachment has also been explored with thermoresponsive, electrospun scaffolds for the culture of C‐MSCs.^109^, ^110^ However, the invasive procedure of suturing the scaffold to the ocular surface seems unfavorable compared to nonsurgical alternatives.

## 8 CONCLUSION

This review highlights important factors that must be considered when developing topical MSC therapies for OSIDs, including stem cell type and source; cell culture; and the choice of substrate for topical application. There is an abundance of data demonstrating the key role of inflammation in the pathogenesis of OSIDs, the awareness of MSC potent immunomodulatory capacity, and the advancements in bioengineering of scaffolds for application to the ocular surface. However, there is limited research which incorporates all this information together to treat ocular surface disorders. Although further research is required, a topical immune‐modulating, stem cell therapy for OSIDs appears to be feasible and exciting.

## CONFLICT OF INTEREST

L.E.S. declared intellectual property rights and research funding from NuVision Biotherapies Ltd. The other authors declared no potential conflicts of interest.

## AUTHOR CONTRIBUTIONS

L.J.B.: conception and design, collection and assembly of data, data analysis and interpretation, and manuscript writing; D.S.J.T.: data analysis and interpretation and manuscript writing; L.S.: conception and design, manuscript writing, and final approval of manuscript.

## Data Availability

Data sharing is not applicable to this article as no new data were created or analyzed in this study.
